# Infantile ZFTA Fusion–Positive Tumor of the Posterior Fossa: Molecular Tumor Board

**DOI:** 10.1200/PO.22.00226

**Published:** 2023-03-02

**Authors:** Vera A. Paulson, Yajuan J. Liu, He Fang, Sam R. Browd, Jason S. Hauptman, Jason Wright, Christina M. Lockwood, Sarah E. S. Leary, Bonnie L. Cole

**Affiliations:** ^1^ Department of Laboratory Medicine and Pathology, Genetics and Solid Tumor Laboratory, University of Washington, Seattle, WA; ^2^ Department of Laboratory Medicine and Pathology, Clinical Genomics Laboratory, University of Washington School of Medicine, Seattle, WA; ^3^ Division of Neurosurgery, Department of Neurological Surgery, University of Washington, Seattle Children's Hospital, Seattle, WA; ^4^ Radiology, Seattle Children's Hospital, University of Washington; Seattle, WA; ^5^ Cancer and Blood Disorders Center, Seattle Children's Hospital; Department of Pediatrics, University of Washington; Center for Clinical and Translational Research, Seattle Children's Research Institute, Seattle, WA; ^6^ Fred Hutchinson Cancer Research Center, Seattle, WA; ^7^ Department of Laboratories, Seattle Children's Hospital, University of Washington; Seattle, WA; ^8^ Brotman Baty Institute for Precision Medicine, Seattle, WA

## CASE

**FIG 1.**
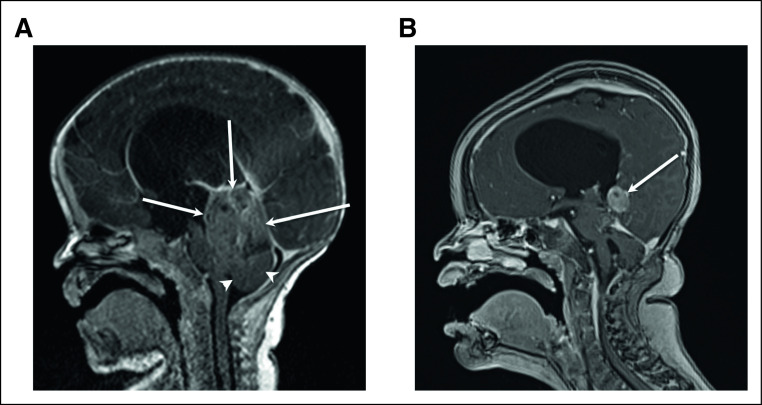
Magnetic resonance imaging of the tumor. (A) Sagittal contrast enhanced T1-weighted image at presentation at age 2 months demonstrated a large avidly enhancing mass situated in the quadrigeminal plate cistern of the posterior fossa (arrows), with superior displacement of the tentorium and straight sinus and inferior displacement of the cerebellar vermis (arrowheads). (B) Sagittal contrast enhanced T1-weighted image at relapse at age 3.5 years demonstrated a recurrent tumoral nodule with avid contrast enhancement, similar to the primary tumor.

A 2-month-old female child presented with fussiness, macrocephaly, and magnetic resonance imaging demonstrating a fourth ventricular mass associated with obstructive hydrocephalus (Fig 1A). Because of unstable clinical condition and young age, initial surgery was limited to tumor biopsy, which histologically demonstrated an embryonal appearing neoplasm composed of sheets of primitive small round blue cells without perivascular nuclear free zones. Nuclear beta-catenin staining led to the presumptive diagnosis of a WNT-activated medulloblastoma. However, the tested tumor sample was negative for an activating variant in *CTNNB1*, and there was no evidence of monosomy 6 using a targeted DNA-based next-generation sequencing (NGS) panel (OPXv5); see Methods for additional information.^1^ Instead, a variant of uncertain significance was identified in SUFU (p.V148M), suggesting the possibility of a SHH-activated medulloblastoma subgroup. Peripheral blood testing determined that the *SUFU* VUS was germline. Lending further support to a possible familial tumor predisposition syndrome was a family history of basal cell carcinoma in the patient's maternal grandmother. The patient was treated with a modified infant medulloblastoma chemotherapy protocol on the basis of CCG 99703: three induction cycles of methotrexate, cisplatin, vincristine, cyclophosphamide, and etoposide, followed by two consolidation cycles of high-dose carboplatin and thiotepa with stem-cell rescue.^2^ Therapy was complicated by hydrocephalus and systemic cytomegalovirus infection, which was treated with ganciclovir and led to early discontinuation of therapy following two rather than three planned cycles of consolidation. Second-look surgery following four cycles of chemotherapy achieved near-total resection of residual tumor, which was < 1% viable.

**FIG 2.**
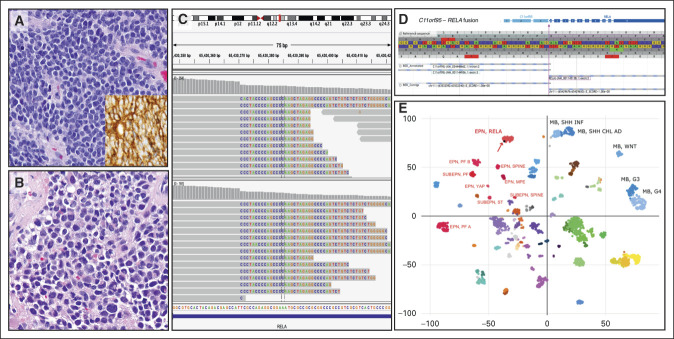
Histologic examination of the initial tumor resection demonstrated (A) classic embryonal features (and L1CAM positivity, inset) identical to (B) those of the recurrence. (C) UW-OncoPlex identified a *ZFTA* (aka *c11orf95*)*-RELA* fusion in both tumor specimens, which was confirmed upon (D) RNA sequencing. (E) Whole genome DNA methylation profiling classified the signature as ST-EPN-RELA.

Routine surveillance imaging 38 months after initial diagnosis revealed tumor recurrence in the vermis, left peritrigonal area, and left tentorial leaflet (Fig 1B). Stereotactic biopsy of the supratentorial left peritrigonal lesion revealed a neoplasm histologically identical to the original tumor (Figs 2A and 2B; Data Supplement). Molecular characterization of the recurrence on an updated DNA-based NGS platform (OPXv6) detected a *ZFTA-RELA* fusion (Fig 2C) in addition to the previously identified *SUFU* VUS.^3^ Fusion identification was discussed at the monthly Pediatric Brain Molecular Tumor Board (MTB) meeting, which is attended by neuro-oncologists, anatomic pathologists, clinical scientists in molecular pathology, radiologists, surgeons, genetic counselors, and support staff. Group consensus was to pursue further characterization of the tumor tissue from the initial biopsy. The initial biopsy was subsequently re-sequenced on OPXv6, evaluated by RNA-based FusionPlex, and evaluated by whole genome DNA methylation profiling using an Illumina 850K MethylationEPIC BeadChip; see Methods for additional details.^3‐5^ These studies confirmed the presence of an in-frame *ZFTA-RELA* fusion with canonical breakpoints (exon 3 to exon 2, respectively; Figs 2C and 2D) and classified the tumor as ST-EPN-RELA with a calibrated classifier score of 0.93 (Fig 2E; Data Supplement).^6^ Additionally, immunohistochemistry performed retrospectively on the initial biopsy was remarkable for L1CAM positivity (Fig 2A, inset; Data Supplement). The current WHO definition of supratentorial ependymoma, *ZFTA* fusion–positive specifically precludes posterior fossa tumors; therefore, the combined features supported a revised diagnosis of “Posterior fossa tumor, *ZFTA* fusion–positive.” Upon completion of these studies, the patient was re-presented at the MTB for further discussion. Ultimately, treatment included craniospinal proton radiation therapy, with a boost to the tumor bed, followed by maintenance chemotherapy including bevacizumab, irinotecan, and temozolomide.^7^ The patient remains alive now nearly 60 months after initial diagnosis and 30 months after relapse.

## METHODS

### Histology

Haemotoxylin and Eosin (H&E)-stained slides of the initial tumor contained a primitive neoplasm growing in sheets with ovoid nuclei and minimal amounts of cytoplasm. Cells had prominent mitotic and apoptotic activity, and perivascular pseudo-rosettes were absent. At the time of original tumor biopsy, tumor nuclei demonstrated retained INI-1 and significant nuclear beta-catenin staining in most tumor cells; this led to an initial presumptive diagnosis of WNT-activated medulloblastoma (see the Data Supplement). Because of limited amounts of biopsy material, no other immunostains were performed in real time. The relapsed tumor subsequently demonstrated similar histology on H&E (see the Data Supplement). As the stereotactic relapse tumor biopsy was quite small, a decision was made to prioritize tissue for molecular testing instead of performing additional immunohistochemical stains. Retrospective staining of the original tumor revealed that tumor cells were strongly positive for L1CAM, essentially negative for synaptophysin, were negative for EMA without areas of dot-like positivity, and were largely negative for GFAP although a subset of tumor cells around vessels stained positive.

### Targeted DNA Sequencing

DNA extracted from formalin-fixed paraffin embedded (FFPE) tissue using the Qiagen GeneRead DNA FFPE Kit (Qiagen, Valencia, CA) was sheared before library preparation using KAPA HyperPrep reagents (Roche, Wilmington, MA). Prepared libraries were hybridized to the UW OncoPlex version 5 (OPXv5, customized Agilent SureSelect probes) or UW OncoPlex version 6 (OPXv6, customized IDT probes), which targets 262 and 340 genes, respectively, as previously described.^1,3^ Libraries were sequenced on Illumina NextSeq500 and HiSeq2500 platforms (Illumina, San Diego, CA) and processed through an automated, custom-designed bioinformatics pipeline developed by the University of Washington NGS Analytics Laboratory.^1,3^

### Targeted RNA Sequencing

cDNA synthesized from FFPE-extracted RNA underwent targeted capture using the Archer custom-designed FusionPlex BBI Solid Tumor Panel and reagent kit (ArcherDX, Boulder, CO). Libraries were sequenced on Illumina NextSeq500 (Illumina Inc, San Diego, CA), and resulting FASTQ files were processed using Archer Analysis software. Human genome build GRCh37 (hg19) was used.^4^

### Whole Genome Methylation Analysis

DNA extracted from FFPE underwent bisulfite conversion using the EZ DNA Methylation Kit (Zymo Research, Irvine, CA). After desulphonation, bisulfite-converted DNA was restored using the NEBNext FFPE DNA Repair Kit (New England Biolabs, Ipswich, MA), amplified, fragmented, and hybridized to the Illumina Infinium MethylationEPIC BeadChip v1.0 (Illumina Inc, San Diego, CA), which features over 850K methylation sites across the genome. After hybridization, the microarray was washed, labeled, stained, and scanned with an Illumina iScan (Illumina Inc, San Diego, CA). Intensity ratio data of the fluorescent signals were generated using iScan Control Software (Illumina Inc, San Diego, CA). Microarray data were analyzed, visualized, and compared with the reference cohort using Heidelberg brain tumor methylation classifier version 11b6, which was based on over 2,800 neuropathologic tumors representing over 80 distinct tumor methylation classes for classification.^5^ Genome build GRCh37 was used.

### Cytogenomic Microarray Analysis

DNA extracted from FFPE was hybridized to an Illumina Infinium CytoSNP-850K BeadChip v1.1 (Illumina Inc, San Diego, CA). After hybridization, the microarray was washed, labeled, stained, and scanned with an Illumina iScan (Illumina Inc, San Diego, CA). Allele and intensity ratio data of the fluorescent signals were generated, and microarray data were visualized and analyzed using Nexus 8.0 (Biodiscovery Inc, El Segundo, CA) to identify chromosomal copy number alterations and regions of copy neutral loss of heterozygosity. Genome build GRCh37/hg19 was used.

Written informed consent was obtained from the parents. This patient's parents signed written informed consent for research use of tissue and medical information under the Seattle Children's institutional banking and biology study, which is approved by the Seattle Children's Institutional Review Board. The parents also specifically consented to subsequent publication of clinicopathologic findings.

## DISCUSSION

In 2016, the WHO Classification of Tumors of the Central Nervous System established the framework for the morpho-molecular diagnosis of brain tumors.^8^ There emerged a new diagnostic entity, “Ependymoma, *RELA* fusion–positive.”^8^ Initially described in 2014, *RELA* fusions appeared to be pathognomonic for a prominent subset of supratentorial ependymomas.^9^ This finding was reinforced following whole genome DNA methylation profiling of a cohort of 500 ependymal tumors, which identified nine subgroups, including the supratentorial ependymoma-RELA (ST-EPN-RELA) subgroup.^10^ More recently, these tumors have been renamed “Supratentorial Ependymoma *ZFTA* fusion–positive” in the updated 2021 WHO Classification of Tumors of the Central Nervous System.^11^ These tumors, however, rarely occur infratentorially.^12^ Also included in 2016 was a major restructuring of the classification of medulloblastoma into one of four distinct subgroups which differed in both underlying biology and clinical characteristics: wingless-activated (WNT), sonic hedgehog-activated (SHH), group 3, or group 4.^8^ Differentiating ependymoma from medulloblastoma is straightforward in most cases; however, rare exceptions occur.

This is the youngest patient ever described with a *ZFTA* fusion–driven neoplasm originating in the posterior fossa.^6^ In 2016, when this tumor was first diagnosed, institutions were in the process of implementing the recently updated WHO classification guidelines incorporating molecular information into tumor diagnoses, including those pertaining to medulloblastoma and ependymoma.^8^ While *ZFTA* fusion–positive tumors are known to occasionally have an embryonal histologic appearance, they are thought to arise in the supratentorial region of the brain. Therefore, the initial molecular testing of this patient's tumor, which did not include *ZFTA* fusion status, was assumed to be sufficient to provide an integrated diagnosis.^8^ The presence of the fusion was incidentally identified three years later during molecular profiling of the patient's recurrence. This case illustrates the necessity of broad, unbiased molecular profiling in pediatric brain tumors and the importance of the MTB in ensuring optimal therapy, particularly in complex and/or unusual cases.

This case also illustrates some of the challenges surrounding the classification of embryonal-appearing tumors in the CNS, which have been well-described.^13,14^ Outside of medulloblastoma, most CNS neoplasms with embryonal morphology are rare, and as such, their classification continues to evolve alongside an understanding of tumor biology. Neuropathologists must be aware of this continuing evolution and also be able to assess key molecular drivers essential for rendering a diagnosis. Additionally, molecular data must be interpreted within the clinical context. Initially, the presence of the *SUFU* VUS, the patient's young age, and the reported family history were suggestive of SHH-activated medulloblastoma and possible carrier status of a cancer predisposition syndrome; to our knowledge, the significance of this variant remains unclear to date.

The response to treatment in this case is also noteworthy. Standard therapy for *ZFTA* fusion–positive supratentorial ependymoma in older children is complete surgical resection followed by focal radiation; currently, the benefit of chemotherapy is being evaluated in a Children's Oncology Group phase 3 clinical trial (ClinicalTrials.gov identifier: NCT01096368). Because of extremely young age, this patient was treated with an intensive chemotherapy regimen following initial biopsy. The tumor responded well radiologically and histologically, with < 1% viable tumor at definitive surgical resection. Although the tumor did recur approximately 3 years later, this interval is longer than might be expected for a typical medulloblastoma recurrence in young children and is also more consistent with the clinical behavior of an ependymoma. Radiation therapy is considered a standard approach to relapsed embryonal tumors and ependymoma while chemotherapy remains controversial in ependymoma. The clinical decision to treat with chemotherapy was made with parental counseling about the incongruency between histologic and molecular diagnoses and based on the expressed wish to treat with curative intent. It is of course not known whether outcome would have been different without chemotherapy, but the patient remains alive now 30 months following recurrence.

In conclusion, this case broadens our understanding of *ZFTA* fusion–positive neoplasms. It also highlights the need for unbiased and broad clinical molecular profiling of pediatric brain tumors, which, at our institution, includes total nucleic acid extraction for all pediatric tumors undergoing molecular characterization. The majority of the tested tumors are then subjected to a targeted DNA-based NGS panel (OPXv6) with the option to reflex to whole genome methylation and/or targeted RNA-based fusion testing following MTB recommendations.

## Data Availability

The data sets used and/or analyzed during the current study are available from the corresponding author on reasonable request.
